# Ultrasound Pretreatment Lymph Node Evaluation in Early-Stage Breast Cancer: Should We Biopsy High Suspicion Nodes?

**DOI:** 10.3390/clinpract13060134

**Published:** 2023-11-24

**Authors:** Mihaela Ionică, Răzvan Ștefan Ilina, Octavian Constantin Neagoe

**Affiliations:** 1 Second Clinic of General Surgery and Surgical Oncology, Emergency Clinical Municipal Hospital, 300079 Timișoara, Romania; razvan.ilina@umft.ro (R.Ș.I.); neagoe.octavian@umft.ro (O.C.N.); 2 Second Discipline of Surgical Semiology, First Department of Surgery, ”Victor Babeș” University of Medicine and Pharmacy, 300041 Timișoara, Romania; 3 Breast Surgery Research Center, ”Victor Babeș” University of Medicine and Pharmacy, 300079 Timișoara, Romania

**Keywords:** fine needle aspiration biopsy, sentinel lymph node, mastectomy, breast conserving surgery

## Abstract

Background: With the growing incidence of breast cancer, efficient and correct staging is essential for further treatment decisions. Axillary ultrasound (US) remains the most common method for regional nodal involvement assessment. The aim of this study was to evaluate whether high-risk US features can accurately predict axillary lymph node metastasis. Methods: A total of 150 early-stage breast cancer patients (T1 or T2) were prospectively included in the study. Based on axillary US, patients were classified as normal, low-risk, or high-risk, with all patients in the low-risk and high-risk groups undergoing fine-needle aspiration (FNAB) and core-needle biopsies. Results: For the low-risk US group, a lower prediction rate of axillary nodal metastasis was achieved than for the group with high-risk features, recording a sensitivity of 66.6% vs. 89.2%, a specificity of 57.1% vs. 100%, a positive predictive value (PPV) of 26.6% vs. 100%, a negative predictive value (NPV) of 88% for both groups, and an accuracy of 58.9% vs. 94%, respectively. FNAB resulted in more false-negative results compared to core-needle biopsy in both low-risk and high-risk US groups. Conclusions: Our findings suggest that high-risk US features can predict axillary lymph node metastasis with high accuracy.

## 1. Introduction

Breast cancer continues to represent the most common type of malignancy diagnosed in women, with an estimated incidence of 2.3 million new cases per year. The burden on healthcare systems is even greater if we consider that breast cancer is also the leading cancer-related death cause in women worldwide [1,2]. With the increasing emphasis on early detection and individualized treatment strategies, accurate staging has become essential in the management of breast cancer patients.

Locoregional lymphatic dissemination remains an important part of breast cancer staging, not only having a significant impact on treatment choice but also representing one of the strongest long-term outcome factors [3]. Traditionally, invasive procedures such as axillary lymph node dissection (ALND) and sentinel lymph node biopsy (SLNB) have been employed for nodal staging. However, these are associated with significant postoperative morbidity, particularly following ALND. Moreover, clinical trials in recent years have shown that similar survival outcomes and regional recurrence rates can be observed in early-stage breast cancer patients with clinically negative or limited metastatic involvement of the axillary lymph nodes who undergo breast conserving surgery (BCS) and radiotherapy (RT), compared to patients who undergo more radical treatment options [4,5,6,7,8]. Therefore, the ability to accurately detect nodal involvement and metastatic axillary extent can help avoid unnecessary surgical procedures, reduce associated morbidities, and optimize individualized treatment plans.

Ultrasound is the most commonly used imaging technique for the evaluation of axillary lymph node involvement. Although no standardization of US criteria exists, several morphologic features have been associated with the metastatic infiltration of axillary lymph nodes. US suspicious lymph nodes undergo fine-needle aspiration biopsy (FNAB) or core-needle biopsy to further aid treatment decisions. More recent advancements in US technique and a better understanding of the US-specific features of metastatic lymph nodes may determine a better selection of patients for minimally invasive procedures.

The present study aims to compare the results of minimally invasive techniques with ultrasound evaluations, with regard to the assessment of axillary lymph node metastasis, in order to assess the efficiency of using only a non-invasive method in the staging of breast cancer patients with a high suspicion of nodal involvement.

## 2. Materials and Methods

This was a single-institution prospective study performed in a high-volume center for breast cancer. The study was approved by our Institutional Review Board, and informed consent was obtained from all patients. A total of 150 women diagnosed with breast cancer between January 2019 and December 2022 were included in the study. Patient files and institutional electronic databases were reviewed for the following clinical information: age, body mass index (BMI), menopausal status, original breast tumor size, axillary lymph node status, and immunohistochemical results for the estrogen receptor, progesterone receptor, human epidermal growth factor receptor 2 status (HER2), Ki-67 proliferation index, and lymphovascular invasion (LVI) for the primary breast tumor biopsy specimens. The inclusion criteria specified female patients aged over 18 years with unifocal breast invasive ductal carcinoma (IDC) confirmed through core-needle biopsy; T1 or T2 breast tumors according to TNM staging; and clinically negative axillary examination (cN0) with luminal A or B molecular subtypes, without neoadjuvant treatment. The exclusion criteria specified incomplete data; multifocal breast cancer; morphologic breast cancer types other than IDC; aggressive molecular subtypes (HER2 positive, triple negative breast cancer); the presence of other synchronous or previous cancers, including previous breast cancer or invasive cancers of the axillary, thoracic, cervical, or superior limb regions; previous oncologic therapies; chronic autoimmune or inflammatory diseases; and reactive lymphadenopathies in the context of acute thoracic, cervical, or superior limb inflammatory disorders.

All patients underwent a preoperative axillary US examination performed with a 12 MHz linear array transducer LOGIQ S7 from GE Healthcare (Milwaukee, WI, USA) by 2 surgeons at our department, with 7 and 20 years of experience, respectively. The axillary US was performed with the patients in an oblique position with the arm raised. All identified axillary lymph nodes were examined for morphologic changes, with the following data being recorded: number of lymph nodes, major diameter, minor diameter, Solbiati index (SI), cortical thickness, lymph hilum aspect, and blood flow. The Solbiati index was measured as a ratio between the maximal longitudinal axis and the transversal axis of the lymph node. Lymph nodes were classified as having a normal, low, or high suspicion of metastasis based on their US aspect, as can be observed in Table 1. Patients were grouped according to these 3 risk classes. Cortical morphologic changes in lymph node structure were classified from type 1 to type 6, based on the classification system proposed by Bedi et al.: type 1, hyperechoic, no visible cortex; type 2, thin hypoechoic cortex (<3 mm); type 3, hypoechoic cortex thicker than 3 mm; type 4, generalized lobulated hypoechoic cortex; type 5, focal hypoechoic cortical lobulation; type 6, totally hypoechoic node with no hilum [9].

All patients with a low or a high suspicion of metastatic nodal involvement underwent both FNAB and core-needle biopsy. All biopsy procedures were performed by the same surgeons who performed the US. Patients with normal US features underwent either SLNB or ALND. Patients with a positive core-biopsy result were reevaluated and restaged, with 5 and 48 patients being referred for neoadjuvant treatment in the low-risk and high-risk groups, respectively. Postoperative pathologic findings were reviewed.

Statistical analysis of continuous data was performed using an independent sample t test or ANOVA, whereas categorical variables were analyzed using a chi-square (χ^2^) test or Fisher’s exact test to identify the US features associated with recurrent lymph node metastases. The overall sensitivity, specificity, accuracy, PPV, NPV, false-positive rate, and false-negative rate for axillary US were calculated. The statistical analyses were performed using SPSS version 19.0 software (IBM Corporation, Armonk, NY, USA). All performed tests were two-sided, and a *p* value of <0.05 was considered statistically significant.

## 3. Results

A total of 120 patients were included in the study. The study cohort was divided into three groups based on the axillary US features, with 40 patients per group. The mean age recorded for the entire group was 48.7 ± 16.2 years (22–78 years), with no statistically significant difference between study groups. Baseline patient and breast tumor characteristics are displayed in Table 2. Overweight and obesity were more prevalent in patients displaying high suspicion criteria on axillary US, whereas patients of normal weight displayed predominantly normal and low US features. Menopausal status and tumor size presented no significant differences among the groups.

With regard to primary breast tumor characteristics, LVI was observed to present statistically significant differences. LVI was present in 78% of patients classified as high-risk for nodal involvement, whereas no LVI was noted in 82% and 64% of cases with normal and low-risk US, respectively.

No significant differences among study groups were observed in the mean number of lymph nodes identified on axillary US.

All examined patients had no clinically evident axillary lymphadenopathy on physical evaluation. Preoperative diagnostic procedures comprised FNAB and core-needle biopsy for all patients with low-risk and high-risk US features. For FNAB in the low-risk group, we observed a sensitivity of only 27.2% and a PPV, NPV, and accuracy of 69.2%, 57.8%, and 60%, respectively, with, however, a specificity of 89.1%. A higher FNAB sensitivity of 60.4% was observed in the high-risk group, with both a specificity and PPV of 100%, but with an extremely low NPV of 9.5%; accuracy was recorded at 62%. Values recorded for core-needle biopsy in both groups presented 100% rates for sensitivity, specificity, and PPV. An NPV rate of 100% and 96.4% was observed in the low-risk and high-risk group, respectively.

Only 26.7% of patients displaying low-risk US features presented metastatic nodal involvement. A statistically significant higher proportion of patients in the high-risk group were diagnosed with axillary lymph node metastasis (*p* < 0.001). Following core-needle biopsy in the high-risk group, 96% of patients presented with a positive result, with only two patients presenting a negative result, as shown in Table 3. Both patients underwent SLNB, with both of them presenting metastatic infiltration on the final pathology report. Postoperative pathology reports revealed the presence of micrometastases in 66.7% of patients from the low-risk group; among these, 75% of cases presented one or two metastatic lymph nodes; only 25% of females recorded three or more affected lymph nodes. For the normal US group, only one patient presented macrometastasis in a single node, whereas micrometastases were observed in one or two lymph nodes for the rest of the cases.

Preoperative US examination of axillary involvement revealed a sensitivity of 89.2%, both a specificity and a PPV of 100%, an NPV of 88%, and an accuracy of 94% for patients displaying high-risk features. Far lower values for the prediction of nodal involvement in the low-risk US group were recorded, with a sensitivity of 66.6%, a specificity of 57.1%, a PPV of 26.6%, an NPV of 88%, and an accuracy of 58.9%.

## 4. Discussion

Axillary lymphatic dissemination is an indispensable element in determining breast cancer stage and, implicitly, in choosing an adapted treatment protocol. Moreover, axillary lymphatic metastases have been shown to represent a strong, independent, long-term prognostic factor [3]. Evaluation of the axillary region for possible elements suggestive of nodal involvement is performed using clinical, imagistic, and invasive means, respectively. The clinical classification of the presence or absence of lymphadenopathies is subjected to both subjective elements of evaluation and adjacent reactivity processes that may overestimate the degree of axillary metastatic spread. The usefulness of this method is strictly confined to extreme cases; it is completely useless for early-stage disease.

Axillary US is the preferred choice of imaging examination for the assessment of nodal involvement in breast cancer. Although other imaging techniques, such as magnetic resonance imaging (MRI) and 18F-fluorodeoxyglucose positron emission tomography/computed tomography (18F-FDG-PET/CT), have been used and have shown similar sensitivity, specificity, and PPV and NPV rates, US still remains the most common means of axillary exploration as it also offers the ability to perform diagnostic procedures [10]. In the case of US, sensitivity and specificity rates have been reported with variable values ranging from 77–85% and 62–80%, respectively [9,10,11,12]. Furthermore, the PPV (36–59%) and NPV (55–95%) of US in detecting malignant axillary lymph nodes depend on the prevalence of nodal metastasis in the patient population being studied. In high-risk populations with a higher prevalence of axillary involvement, the PPV tends to be higher, indicating a greater likelihood of true positive results. Conversely, in low-risk populations, the NPV tends to be higher, suggesting a lower probability of true negative results [9,10,11,13]. This highlights the importance of considering the pretest probability of nodal metastasis and integrating US findings with other clinical and radiological factors.

The main reason for the increased variability in reported rates is a lack of standardized US criteria for lymph node assessment. US images of normal axillary lymph nodes are known to be characterized by an oval shape with smooth and well-defined margins, as well as a slightly hypoechoic and consistently thin cortex, measuring 3 mm or less. Lymph nodes with these criteria have been shown to have a high negative predictive value for excluding metastases. On the other hand, rounded hypoechoic lymph nodes with displacement of fatty hilum, indistinct margins, cortical thickening ≥ 3 mm, and focal or diffuse cortical lobulations have been associated to varying degrees with metastatic infiltration. However, focal cortical bulging, eccentric cortical thickening, or diffuse cortical thickening can be observed in both reactive and metastatic lymph nodes. Lymph node size has been used as a possible indicator of nodal involvement, with the longest diameter >2 cm being considered as a threshold. However, as previous studies have pointed out, lymph node size has little bearing on the likelihood of metastatic infiltration. The Solbiati index has shown greater usefulness in the US evaluation of axillary nodal involvement, with a ratio of <2 being considered an at-risk lymph node. For this reason, in the present study, sonographic morphological changes, in particular the aspect of the lymph node cortex, have been taken into consideration for risk stratification. Bedi et al. have developed a classification of axillary lymph nodes based on US cortical characteristics, ranging from type 1 to type 6, where benign features were classified from 1 to 2, and highly suggestive features for malignancy were classified as 5 or 6. The PPV for types 5 and 6 were reported at 29% and 58%, respectively. However, these rates can be hard to translate into clinical practice, as the US axillary examination was performed ex vivo on surgical specimens following ALND [9].

Other factors outside of US features have been suggested to be taken into consideration for the risk assessment of axillary lymphatic metastasis. LVI signifies the existence of tumor cells within lymphatic spaces or blood vessels, or both, in the peritumoral area. It is an important stage in the invasion metastasis cascade and can be identified through microscopic examination of the primary tumor. LVI has been shown to represent an independent factor for long-term outcome and disease recurrence. Liu et al., in a study for the elaboration and validation of an axillary US nomogram, have shown LVI to represent an independent factor for predicting nodal metastasis in both univariate and multivariate analysis [13]. Lymph node cortical thickness and obliterated hilum were the only two other US features highlighted in their study as representing independent factors predicting axillary lymph node metastasis. The results of our study are in agreement with these findings, as LVI was observed in 78% of patients with high-risk US profiles, with only a small proportion of patients with normal or low-risk US presenting LVI in the primary breast tumor.

In the design of the present study, only patients with IDC were considered. This was due to the less than well-defined US features of nodal involvement observed in other histological types of breast cancer. Studies on the US characteristics of metastatic axillary lymph nodes from lobular carcinoma have described a diffuse cortical thickening without displacement of the fatty hilum, decreasing US sensitivity and leading to a higher false-negative rate [14]. These aspects may also impact FNAB results, as cytology reports are more likely to yield a false-negative result when metastatic infiltration of the node is less than 30% or when cortical thickness is less than 3.5 mm [15]. Lower thresholds for US features of lymph nodes and use of core-needle biopsy could be considered as alternative measures in these patients for establishing a proper pretreatment evaluation.

The recommendations of international guidelines for performing axillary surgery in breast cancer have seen significant change over time. During the 1970s, pivotal discussions arose, and two significant trials, the Kings/Cambridge and NSABP-04 trials, challenged the conventional wisdom of the modified radical mastectomy. These trials involved the randomization of patients with clinically negative axillary nodes into groups receiving early or delayed axillary treatment [16,17]. By the mid-1980s, the landscape of axillary surgery had begun to evolve, moving towards a less aggressive approach [18,19]. This shift was underpinned by a historical perspective, culminating in the 1990s with a momentous transition from ALND to the adoption of SLNB [20,21,22]. The introduction of SLNB marked a significant scientific advancement, particularly benefiting women with early-stage breast cancer.

Over the past few decades, there has been a growing recognition that axillary surgery serves primarily as a tool for staging and prognosis assessment, rather than as a therapeutic intervention. In the 1990s, several trials comparing SLNB with ALND consistently demonstrated that SLNB was associated with a lower morbidity and a higher quality of life, without impact on overall survival [20,22,23]. An even less aggressive surgical approach in the treatment of early-stage breast cancer has been adopted in the past 10 years, from ALND to SLNB and, more recently, to only FNAB or core biopsy. Among the initial studies to introduce and support this concept was the ACOSOG-Z0011 trial. The Z0011 trial randomized early-stage breast cancer patients who presented with 1–2 positive lymph nodes following SLNB to receive ALND or no further surgical treatment. The findings of the study were surprising, showing no benefit in performing a more aggressive surgical treatment, with an approximate 1% axillary relapse at 6.3 years of follow-up [4]. Based on these results, the European Institute of Oncology in Milan started in 2012 the SOUND (sentinel node vs. observation after axillary ultrasound) study, a prospective noninferiority phase 3 randomized clinical trial, performed as a multicentric analysis in Italy, Switzerland, Spain, and Chile on a total of 1463 women with early-stage breast cancer. The authors proposed an observational strategy based on axillary US in early-stage breast cancer rather than a surgical approach [24]. Earlier this year, the results of the SOUND trial were published, demonstrating that the omission of axillary surgery resulted in similar outcomes to those experienced by patients undergoing SLNB [25]. Large trial studies, such as IBCSG 23-01 and AMAROS, have demonstrated no benefit to survival or long-term outcomes for patients who undergo radical ALND compared to patients who receive BCS and local RT, demonstrating good disease control not only as regards the management of micrometastases but also in the cases of patients with metastatic spread limited to one or two lymph nodes [4,5,6]. These findings have not only changed current treatment guidelines; they have also opened a new perspective on the therapeutic approach to early-stage breast cancer [3]. Ongoing trials, such as the NAUTILUS study, explore the possibility of omitting SLNB in breast cancer patients who are candidates for BCS and have a clinical and US negative axilla [11]. Current guidelines, such as those published by the European Society for Medical Oncology (ESMO), recommend SLNB in favor of ALND as a standard of care in patients with early-stage breast cancer and clinically node-negative axillae. Further surgical treatment is not required in cases of positive SLNBs with a low disease burden, such as micrometastases or up to two metastatic lymph nodes, with axillary RT being considered as a valid alternative. Following the same line, the National Comprehensive Cancer Network (NCCN) guidelines recommend SLN mapping in all patients with clinically or imaging negative axillary involvement or ≤2 lymph nodes that are suspicious on imaging or positive on biopsy. No further axillary surgery is recommended if micrometastases are observed on SLNB. Similarly, ALND is not indicated for patients with cT1-2, with one or two positive lymph nodes on SLNB, who undergo no preoperative chemotherapy and for whom adjuvant RT is planned.

Our study proposes that patients presenting with high-risk US features of nodal involvement are to be considered positive without the need for preoperative biopsy procedures. In our sample, 96% of US high-risk patients were confirmed to have metastatic lymph nodes on core-needle biopsy; however, both patients who recorded a negative result were confirmed to have metastatic axillary lymph nodes in the final pathology report. This could be explained by a possible error in sampling technique. As previously mentioned, primary breast tumor characteristics, such as LVI and aggressive molecular subtypes, are more commonly associated with high-risk US features. As such, the sensitivity and specificity of US in these patients could also be increased by taking breast tumor characteristics into consideration. In addition, tissue sampling techniques remain essential in the assessment of patients with low-risk US features. The proper evaluation of suspicious nodes is important, as patients with limited nodal involvement may be candidates for local RT rather than SLNB or ALND. FNAB and core-needle biopsy are the usual techniques for axillary tissue sampling, but with certain limitations. FNAB is reported to exhibit variable sensitivity and specificity rates, presenting with limited NPV and false-negative results. FNAB has been shown to possess lower rates in all aspects, including accuracy and the amount of obtained tissue sample compared to core-needle biopsy [26,27,28,29,30]. In the same area, data from our series have shown 100% rates for sensitivity, specificity, PPV, and NPV for patients with both low-risk and high-risk US features. Although more invasive than FNAB, core-needle biopsy offers a more reliable evaluation of nodal metastatic spread. However, FNAB remains a safer alternative for patients with lymph node locations that are not eligible for core-needle biopsy.

Furthermore, artificial intelligence systems are being developed for the purpose of increasing US detection rates of lymphatic metastasis. Deep learning algorithms are frequently utilized in image prediction and diagnosis due to their advantages in terms of speed, accuracy, and reproducibility. These algorithms have the potential to identify features in clinical images that human specialists often overlook and can provide remote quantitative estimates [31]. Ashokkumar et al. propose a deep learning model for the prediction of metastatic axillary lymph nodes in patients with breast cancer, with a reported sensitivity of 93–98%, a specificity of 93–99%, and a receiver operating curve of 0.94–0.95, substantially outscoring expert radiologists [31]. The use of such systems in the evaluation of clinically lymph node-negative breast cancer patients may further limit the need for invasive diagnostic procedures.

The limitations of this study must be acknowledged. Firstly, the study has taken into consideration several exclusion criteria that may restrict the generalization of our research, such as only IDC type breast cancer patients being included, limiting the possibility of extending current findings to other forms of breast cancer. Secondly, some bias may exist as the study sample is relatively small, and data were collected from a single institution and are therefore not fully representative of the entire population. Further studies on larger patient samples are ongoing, and external validation through independent cohorts is still necessary.

## 5. Conclusions

In conclusion, in selected patients with T1/T2 IDC type breast cancer, a high suspicion on axillary US is accompanied by a high sensitivity and specificity for metastatic involvement; this reduces the need for invasive diagnostic procedures, aiding staging and ulterior treatment decision planning.

## Figures and Tables

**Table 1:** Axillary US criteria for lymph node metastasis.

Classification	US Characteristic
Normal	Oval shape, uniform cortex < 3 mm (type 1 or 2), smooth margins, SI > 2
Low suspicion	Focal or diffuse cortical thickness > 3 mm (type 3 or 4), presence of increased peripheral blood flow
High suspicion	Focal or complete hypoechoic cortex (type 5 or 6), round shape, SI < 2, complete or near complete loss of fatty hilum, indistinct margins

**Table 2:** Study group baseline characteristics.

Characteristics	Normal *N* (%)	Low Suspicion *N* (%)	High Suspicion *N* (%)	*p* Value
Age (years)	47.3 ± 12.3	49.8 ± 17.1	51.7 ± 14.4	n.s.
BMI (kg/m^2^)				
<25	27 (67.5%)	22 (55%)	14 (35%)	<0.02
≥25	13 (32.5%)	18 (45%)	26 (65%)	
Postmenopausal				
Yes	19 (47.5%)	17 (42.5%)	24 (60%)	n.s.
No	21 (52.5%)	23 (57.5%)	16 (40%)	
Tumor size				
T1	11 (27.5%)	14 (35%)	9 (22.5%)	n.s.
T2	29 (72.5%)	26 (65%)	31 (77.5%)	
Molecular subtype				
Luminal A	28 (56%)	31(62%)	21 (42%)	n.s.
Luminal B	22 (44%)	19 (38%)	29 (48%)	
LVI				
Yes	9 (18%)	18 (36%)	39 (78%)	<0.001
No	41 (82%)	32 (64%)	11 (22%)	
No. of US identified lymph nodes	1.7 ± 1.4	2.7 ± 1.1	2.5 ± 1.7	n.s.

BMI—body mass index; LVI—lymphovascular invasion observed in primary breast tumor biopsy specimen; US—ultrasound; n.s.—not statistically significant.

**Table 3:** Distribution of lymph node evaluation using US and pathology examination.

US Risk Class	FNAB *N* (%)		Core-Needle Biopsy *N* (%)		Postoperative Pathology *N* (%)	
	Benign	Malignant	Benign	Malignant	Benign	Malignant
Normal	–	–	–	–	44 (88)	6 (12)
Low-risk	37 (74)	13 (26)	33 (66)	17 (34)	33 (73.3)	12 (26.7)
High-risk	21 (42)	29 (58)	2 (4)	48 (96)	0	2 (100)

Values for FNAB and core-needle biopsy of patients with normal axillary US are absent, as these patients did not undergo any invasive preoperative axillary procedures.

## Data Availability

Data are available on request.
